# Perceptions of SNAP and Stocking Standards: A Qualitative Study of California Small Food Store Owners and Managers

**DOI:** 10.3390/nu13030752

**Published:** 2021-02-26

**Authors:** Anthony Meza, June M. Tester, Irene H. Yen, Barbara A. Laraia, Julia A. Wolfson, Cindy W. Leung

**Affiliations:** 1 Undergraduate Program, School of Public Health, University of California, Berkeley, CA 94704, USA; ameza@berkeley.edu; 2 Department of Pediatrics, University of California, San Francisco, Benioff Children’s Hospital Oakland, Oakland, CA 94609, USA; june.tester@ucsf.edu; 3 School of Social Sciences, Humanities, and Arts, University of California, Merced, CA 95343, USA; iyen@ucmerced.edu; 4 Division of Community Health Sciences, School of Public Health, University of California, Berkeley, CA 94704, USA; blaraia@berkeley.edu; 5 Department of International Health, Johns Hopkins Bloomberg School of Public Health, Baltimore, MD 21205, USA; jwolfso7@jhu.edu; 6 Department of Nutritional Sciences, School of Public Health, University of Michigan, Ann Arbor, MI 48109, USA

**Keywords:** community-based participatory research, food insecurity, nutrition policy, poverty, urban health

## Abstract

The Supplemental Nutrition Assistance Program (SNAP) is critical to alleviating food insecurity, but low diet quality among program participants is a concern. Nutrition-related interventions have focused on SNAP-authorized food retailers, but the perspectives of small food store owners and managers have not been represented in national policy discussions. This study aimed to explore the opinions of store owners/managers of SNAP-authorized small food stores about their overall perceptions of the program and the stricter stocking standards previously proposed in 2016. We conducted in-depth, semi-structured interviews with 33 small food store owners and managers in San Francisco and Oakland, California in 2016. Interviews were analyzed for thematic content using the general inductive approach. Four themes emerged from owners/managers’ discussion of their overall perceptions of SNAP: the beneficial impact of SNAP on their business, how SNAP enables them to connect with the broader community, the importance of SNAP in preventing hunger, and the nutrition-related struggles that SNAP participants face. Store owners/managers had a generally favorable response towards the proposed stricter stocking standards. Additional themes discussed pertained to the concern about whether stocking changes would lead SNAP participants to purchase more healthful food and some logistical challenges related to sourcing and storing perishable foods.

## 1. Introduction

The Supplemental Nutrition Assistance Program (SNAP), formerly the Food Stamp Program, is the largest of the federal nutrition assistance programs, reaching 35.7 million low-income Americans in 2019 [1,2]. Although SNAP is critical to alleviating food insecurity [3], diet quality among SNAP participants is consistently lower than that of low-income, SNAP-eligible non-participants [4,5]. Thus, efforts to promote better nutrition within SNAP may improve its effects on both food insecurity and dietary intake for the millions of low-income Americans who rely on it.

Nutrition-related interventions have focused on SNAP-authorized food retailers, which represent a network of approximately 260,000 food stores, including supermarkets, grocery stores, superstores, convenience stores, farmer’s markets, and more [6]. Building on previous efforts to increase the emphasis on nutrition within SNAP [7,8,9], in 2016, USDA proposed that SNAP-authorized retailers be required to stock a greater variety of perishable and non-perishable foods across four staple food groups: (1) fruits and vegetables; (2) dairy; (3) meat, poultry and fish; (4) breads and cereals, and a greater number of each food item [10]. Since the proposal was introduced, USDA subsequently enacted multiple changes that effectively relaxed the stocking criteria back to the original guidelines, in part due to concerns that the proposed stocking requirements would result in an undue burden for small food stores [11,12,13]. However, without empirical data to support this claim, it is difficult to understand the unique challenges that small food stores would face in increasing the availability of healthful food across diverse geographic settings. 

Although the majority of SNAP benefits are redeemed at supermarkets and supercenters, small food stores comprise over 45% of all SNAP-authorized retailers and are fundamental to the local food environment, particularly in low-income communities and areas without large grocery stores [14,15]. The owners and managers of small SNAP-authorized food retailers are important stakeholders in the ongoing discussion regarding improving the nutritional landscape for low-income families receiving SNAP; however, their voices have not been typically represented in national policy discussions. The objective of this study was to explore the opinions of store owners and managers of SNAP-authorized small food stores in two large California cities about their overall perceptions of SNAP, and their attitudes towards the stricter stocking standards proposed by USDA in 2016 including any concerns/challenges they would face upon implementation. 

Proposed changes to SNAP stocking standards need to be feasible, sustainable, and garner broad support across multiple groups of stakeholders if they are to be effective. Although the proposed policy in 2016 has been tabled, similar proposals are likely to arise in the future in the ongoing national discussion to improve the delivery of SNAP. A clear understanding of the perspectives of store owners and managers can inform how future policy proposals may impact SNAP-authorized small food stores. 

## 2. Materials and Methods

### 2.1. Recruitment and Data Collection

Participants were store owners and managers of SNAP-authorized small food stores in San Francisco and Oakland, California. SNAP-authorized small food stores were selected using North American Industry Classification System (NAICS) codes of 445110 (supermarkets and other grocery) and 445120 (convenience stores) from an InfoUSA database. The list of stores was further narrowed to include businesses with a single location, having <10,000 square feet, and with fewer than 10 employees. The InfoUSA database was also limited to the top five zip codes in San Francisco and Oakland with the highest proportion of SNAP households using data from the 2014 American Communities Survey. In San Francisco, these zip codes were 94102, 94103, 94124, 94130, and 94134, ranging from 8.6 to 26.8% SNAP households. In Oakland, these zip codes were 94601, 94603, 94605, 94606, and 94607, ranging from 14.4 to 21.3% SNAP households. Stores were then cross-matched to the USDA SNAP vendor database. In total, 30 stores were eligible from San Francisco and 28 stores were eligible from Oakland. 

Two research assistants from the University of California, San Francisco were trained in qualitative interviewing through didactic and hands-on approaches and conducted all interviews in the present study. Individuals who self-identified as the store owner/manager were recruited in person at their respective store locations. The interviewers followed a standardized recruitment script that described the purpose of the study. If the store owner/manager expressed interest, the researchers confirmed that their store was SNAP-authorized, that they had managed or owned the store for one year, and that they were able to complete an interview in English or Spanish. Verbal consent to audio-record the interview was also obtained. In-depth interviews were conducted in a semi-structured format, allowing the interviewers to delve deeper into the specific questions based on the participants’ responses, as well as respond to new issues related to SNAP during the interview. All interviews were conducted by the same interviewers and were completed in person at the store location. Participants received a $20 Target gift card for their participation. Across all 58 eligible stores in the sample, 10 store owners/managers were unavailable to be interviewed (after five or more attempts), 5 store owners/managers were excluded due to language barriers, and 10 store owners/managers declined to participate in the study, yielding a total sample of 33 stores.

An open-ended interview question guide was adapted from a prior project assessing the attitudes and perspectives of Special Supplemental Nutrition Program for Women, Infants, and Children (WIC) store managers in response to the revised WIC food package [16]. The final interview guide consisted of 17 questions that assessed their general perceptions of SNAP, strategies that SNAP can assist low-income families in eating better, their perspectives on the proposed federal changes to increasing stocking requirements of staple foods for current SNAP vendors, and any challenges they may face in meeting the proposed guidelines. The final interview guide was also translated into Spanish by two native Spanish speakers. 

All interviews were conducted between May and September 2016. Interviews were conducted at their respective businesses, in English or Spanish (according to participant preference) and ranged from 20 to 30 min. The study’s methodology was approved by the University of California, San Francisco Institutional Review Board.

### 2.2. Data Analysis

Interviews were transcribed verbatim from audio recordings and were checked for accuracy. Spanish interviews were translated into English prior to analysis. Transcriptions of the transcribed (and translated) interviews were analyzed for thematic content using the general inductive approach [17]. A coding scheme of themes and subthemes was developed by the Principal Investigator (C.W.L.) for each question and subsequently modified through an iterative process until a final codebook was achieved that encompassed all completed questions. Three project team researchers then independently coded each transcript and reviewed the final transcripts together to reconcile any discrepancies until consensus was reached. The transcripts were coded based on broad categories of themes mentioned in the interview guide as well as major topics that were consistently discussed. The final transcripts were entered into NVivo (QSR International Pty Ltd, version 11, 2017) to assist in organizing the themes and examine whether any patterns emerged by participants’ responses.

## 3. Results

Characteristics of the stores in the study are shown in Table 1. Of the 33 stores, 19 stores were located in San Francisco and 14 stores were in Oakland. Thirteen were small grocery stores and 20 were convenience stores. Utilization of Electronic Benefit Transfer (EBT) cards varied widely across stores interviewed, with 21% of stores reporting <10%, 42% of stores reporting 10–50%, and another 21% of stores reporting >50% of transactions with EBT.

### 3.1. Overall Perceptions of SNAP 

Four themes emerged from the store owners’ and managers’ discussion around their overall perceptions of SNAP: (1) the beneficial impact of SNAP on their business, (2) how SNAP enables them to connect with the broader community, (3) the importance of SNAP in preventing hunger, and (4) the nutrition-related struggles they observed among of the participants who shop in their store. 

The first theme that emerged was the beneficial impact of SNAP on small food stores. Store owners/managers discussed how participating as a SNAP vendor increased their store’s profits. Many were enthusiastic in acknowledging the importance of SNAP in bringing customers to their store, indicating that their sales would be 20–30% lower without participating in SNAP. One San Francisco respondent described SNAP as an opportunity to accept more customers: “Every square footage (of my store) costs money. It affects something. If I don’t accept the EBT card, they are going to go somewhere else, so why miss my opportunity? Because this is where they purchase (their food).” This sentiment was also echoed by an Oakland respondent who said, “Most of the neighborhood has food stamps. They will go somewhere else if we don’t accept food stamps. You just have to (accept it) in this neighborhood.” 

The second theme was the role of SNAP in allowing small food stores to connect with the broader community. Respondents described getting to know the food preferences of their regular customers, and stocking food items due to specific requests from SNAP customers. An Oakland respondent described making special deals when he knew SNAP customers would be more likely to shop as his store: “I see (my customers) five to ten times each day, all of them. They’re my neighbors.” Another San Francisco respondent said, “We like to help people here. You can see they’re my type of people; you feel like you would help them. Whatever they order from me, I will voluntarily get it. I am sure in this neighborhood, you can help a lot of people.” 

The third theme was how they perceived the importance of SNAP in providing food and preventing hunger. Store owners/managers discussed how SNAP enabled people within their communities to purchase food. One San Francisco respondent said, “(SNAP) allows them to buy food and allows them to eat. I think (SNAP) is good because it helps those who don’t have cash available and helps them buy stuff for their families.” Store owners/managers also discussed their awareness of when their customer’s SNAP benefits were replenished, which foods they were most likely to purchase with SNAP benefits, and the different types of families who use SNAP benefits at their store. One Oakland respondent said, “A lot of families get help from EBT, of course. I see a lot of families around here. They only have EBT. They come here and they buy (food) for the kids…cereals, milk, all of these for the health of the kids.”

The final theme revolved around their customers’ struggles to obtain nutritious food. Respondents widely acknowledged that SNAP benefits were sometimes inadequate for SNAP participants to afford enough food for their families. One Oakland owner/manager said, “Some people that come here are not able to work. They basically rely on food stamps to maintain their health. Sometimes it’s not enough, and it’s painful to see. We try our best to help folks here.” Other store owners/managers described the need for more nutrition education targeting SNAP participants to encourage purchases of healthier food. One San Francisco owner/manager said, “Put families in classes and show them which is better for their kids, for their family to eat. Send them letters, explain to them how their health will be much better if they eat (healthier) foods.”

### 3.2. Perceptions of Stricter Stocking Standards and Potential Challenges

The majority of store owners/managers believed that the stricter stocking standards proposed by the USDA were a good idea. While some owners/managers believed their stores already carried sufficient quantities of healthier foods to comply with the USDA proposal, several others expressed willingness to make additional changes to comply with the stricter standards while also noting some of the challenges they may encounter in doing so. Their perceptions towards the stricter stocking standards were summarized across four themes: (1) the proposed standards would benefit their customers; (2) carrying additional healthful foods would result in higher profits; (3) the concern about whether SNAP participants would purchase a greater array of healthful food; and (4) the logistical challenges they would face in sourcing and selling additional perishable food items.

The first theme pertained to the store owners/managers perceptions that carrying more healthful items would allow them to offer more products to their customers and provide a bigger focus on healthful foods, yielding a benefit for their customers. As one San Francisco respondent described, “If the program required (stores) to carry healthier foods, people would be exposed to them more often. It would make it more likely for people to purchase it using (SNAP) due to the exposure of it. People don’t want to go out of their way to go get (healthy food), but if it is in front of them, they would be more likely to eat it.” This sentiment was shared by an Oakland respondent, who said, “There should be more products like fruits and vegetables and stuff like this available to the people. I feel it could help them to eat better and helps us as well.” 

In a similar vein, the second theme focused on the positive impact that carrying more healthful foods could have on their store’s profits. A San Francisco respondent said, “(I would make the changes) if it was required. I don’t think it would be a problem and it would be a good opportunity for profit.” Owners/managers generally believed these changes were an accepted part of being a SNAP vendor, and would help them retain their base of SNAP customers. One Oakland respondent said, “No problem, why not? If I make some money, right? We have a lot of EBT customers. We care about EBT because we have a lot of money from EBT.”

Despite their overall positive attitudes, a third theme involved some concern that SNAP participants would not buy the additional foods even if the stores increased their stock, saying that it was ultimately up to the customers to decide if they wanted to eat healthier. One San Francisco respondent said, “(The new standards are) good…But, it depends if the customers are willing to buy it. We have fruit and vegetables here—look, nice apples, top of the line—nobody buys it. Carrots too—we put here, a stack. Nobody buys it.” Other respondents discussed their beliefs that SNAP was already a good program and its recipients will buy what they want without being “forced” into buying healthier food. As one Oakland respondent said, “They make the change—the customer. The customer is going to choose what (they’re) going to buy or not.”

The final theme centered around the logistical challenges of sourcing and storing additional food, particularly perishable items. One San Francisco respondent discussed the challenge of getting food deliveries given the lack of parking on busy streets. To source his produce, he traveled to the produce market before opening his store. To comply with stricter standards, he would have to make additional trips to meet the increased demands for perishable foods. Other challenges discussed included limited space to store or display food. One Oakland respondent who owned two small food stores said, “We have another business and that one would probably need to have more space, maybe bring (in) more machines. If you were required to have more fruits and vegetables, they would have to be in the refrigerator.” Store owners/managers also described their concerns with storage and space within the confines of being a convenience store. As one San Francisco respondent described, “Similar challenges would all be about space and time, as well as the cost of maintenance and storage. That’s usually a deciding factor with a lot of stores—whether they want to be a (convenience store) or a store known for their produce.”

## 4. Discussion

In this study, San Francisco and Oakland store owners and managers discussed the beneficial impact of SNAP on their business, how SNAP is a link between their store and the broader community, the importance of SNAP in preventing hunger, and the nutrition-related challenges of the participants who shop in their store. Specific to the stocking standards proposed by USDA, store owners/managers expressed overall support for initial guidelines, but also discussed their concerns about whether SNAP participants would purchase a greater array of healthful foods and the logistical concerns they might face in sourcing and selling certain food items. 

This research builds on prior qualitative studies on the perspectives of small food store owners on proposed changes to SNAP stocking standards. Karpyn and colleagues interviewed small food store owners/managers in four states [18]. Their study found “reluctant willingness” among owners/managers to meet the proposed guidelines, with the majority believing they had already met or were close to meeting the new guidelines. Similar to the present study, some store owners/managers believed the changes would increase their sales and positively impact the broader community, whereas other store owners/managers raised concerns about perishable foods spoiling quickly, and the need for nutrition education to encourage SNAP participants to purchase healthier foods. Another qualitative study focused on rural store owners from six states [19]. In these interviews, store owners expressed stronger concerns about the logistical challenges to implementing the proposed guidelines, particularly around sourcing specific products, storage space, equipment, and perishability of foods, and the potential reduction on their profit margins. These viewpoints were in contrast to the store owners/managers in the present study who viewed the changes as financially beneficial, which may be due to the difference in rural versus urban contexts of the studies. All of these viewpoints are important, and demonstrate the mixed reactions from small food store owners/managers to stocking additional healthful foods as a SNAP-authorized retailer. 

Since the interviews in the present study were conducted, substantial changes were made to the proposed stocking guidelines, in part because due to comments received by USDA during a public comment period. Haynes-Maslow and colleagues conducted a content analysis of a random sample of comments and found a stark disconnect between commenters from health and non-profit organizations, education, and private citizens, and commenters from retail/business [20]. Comments from the former groups showed broad support for the rule, stating that the stricter standards would lead to improved healthy food access, improved nutrition, and improved health outcomes. Comments from the retail/business group were opposed to the rule and discussed concerns regarding SNAP retailer rule definitions, stores withdrawing from SNAP, and the increased burden placed on businesses to comply with the new inventory requirements. In general, these concerns were in the minority compared to the positive reactions and willingness to comply that most San Francisco and Oakland store owners/managers expressed. It should also be noted that a majority of the comments received by USDA were retailer template comments containing boilerplate language provided by large retailer chains, which may not reflect the specific sentiments of individual store owners or managers.

Drawing parallels from the 2009 WIC (Special Supplemental Program for Women, Infants, and Children) food package revision, which required WIC-authorized retailers to stock additional healthy food, evidence has emerged demonstrating how federal nutrition policies can improve the local food environment. In a study in New Orleans, authors noted greater availability of lower-fat milk, whole grains, fruits, and vegetables at WIC stores after the policy change [21]. Another study demonstrated similar changes with the greatest improvements found in corner stores and in census tracks with >60% Black residents, suggesting that policies that improve healthy food access may also reduce health disparities [22]. The stocking changes at the store level also resulted in improvements in dietary quality among WIC beneficiaries [23,24]. Despite the different nutritional standards between WIC and SNAP, similar changes to SNAP stocking standards may also yield similar benefits to those seen after the WIC food package revision. A recent study found that approximately 70% of SNAP-authorized small food stores in low-income communities across seven states did not meet some or all of the stocking standards initially proposed by USDA [25]. Thus, revised SNAP stocking standards could be an effective policy mechanism to increase healthy food availability in low-income and minority racial/ethnic communities, and reduce nutrition-related disparities among the individuals receiving SNAP. 

One of the limitations of this study is that the interviews were conducted in two urban cities in California, which may limit the generalizability of the findings. However, the food environments in San Francisco and Oakland are notable in that they are “food swamps” (i.e., areas with a high abundance of unhealthy food stores), the stores are predominantly run by immigrant families, and the stores also serve a diverse clientele due to the high walkability of the neighborhoods located within the zip codes surveyed [26]. Thus, the perspectives of the store owners/managers in the present study are unique from those presented in prior qualitative studies and from the set of public comments received by USDA. The more positive attitudes expressed by store owners/managers in the present study might also be attributed to citywide healthy corner store initiatives or to social desirability bias given that interviews were conducted in a public setting. Another limitation is that the present study summarized the perceptions of a potential policy proposal from small food store owners/managers rather than examining the real-world impact that such a policy might have on supply chains, wholesale purchasing, and other challenges to related to restocking in small food stores. Further research is needed to examine how the economics of these multiple processes compare between small and large food stores and to understand how small changes to a store’s inventory might impact customers’ shopping behaviors.

## 5. Conclusions

In conclusion, the results of this study demonstrate that owners/managers were generally supportive of making changes to comply with stricter stocking standards for healthful foods. Despite knowing there could be some logistical difficulties, the owners/managers generally did not think these were insurmountable hurdles. This study contributes to a growing evidence base on how SNAP-authorized small food stores would perceive future USDA proposals on changing stocking requirements, and underscores the need for federal initiatives to support small businesses in improving healthful food access that would simultaneously benefit SNAP participants.

## Figures and Tables

**Table 1:** Characteristics of small food stores.

Store Characteristic	Number of Stores	Proportion (%)
City		
San Francisco	19	57.6
Oakland	14	42.4
Store type		
Convenience store	13	39.4
Small grocery store	20	60.6
Interview language		
English	30	90.9
Spanish	3	9.1
Number of Full-Time Employees		
0 employees	1	3.0
1–2 employees	14	42.4
3–5 employees	14	42.4
6–10 employees	3	9.1
More than 10 employees	1	3.0
Number of Part-Time Employees		
0 employees	17	51.5
1–2 employees	11	33.3
3–5 employees	4	12.1
6–10 employees	1	3.0
Percentage of transactions with EBT		
<10%	7	21.2
10–25%	6	18.2
26–50%	8	24.2
More than 50%	7	21.2
Don’t know/ Refused	5	15.2

## Data Availability

The data from the present study are available from the corresponding author upon reasonable request.
